# Quantifying setup uncertainty between computed tomography guidance and magnetic resonance guidance in intramuscular metastases radiotherapy

**DOI:** 10.1002/acm2.70589

**Published:** 2026-04-19

**Authors:** Travis Salzillo, Surendra Prajapati, Zhiqian Henry Yu, Yao Zhao, Tze Yee Lim, He Wang, Xin A. Wang, Xinru Chen, Rajat Kudchadker, Belinda Lee, Angela Sobremonte, Ahsan S. Farooqi, Devarati Mitra, Andrew Bishop, Chad Tang, Jinzhong Yang

**Affiliations:** ^1^ Department of Radiation Physics The University of Texas MD Anderson Cancer Center Houston Texas USA; ^2^ Department of Radiation Oncology The University of Texas MD Anderson Cancer Center Houston Texas USA; ^3^ Department of Genitourinary Radiation Oncology The University of Texas MD Anderson Cancer Center Houston Texas USA

**Keywords:** image‐guidance, MR‐Linac, oligometastasis, radiotherapy, treatment margins

## Abstract

**Background and purpose:**

MR‐Linac (MRL) is well‐suited for treatment of oligometastatic disease (OMD) in muscle due to its superior soft tissue contrast. The purpose of this study was to assess dosimetric differences and quantify margins when CT‐guidance (CTgRT) is used for OMD treatment compared to MR‐guidance (MRgRT) on the MRL.

**Materials and methods:**

Five patients with intramuscular oligometastasis were treated with the MRL using daily MRgRT. To simulate CT‐based alignment, an in‐house deep learning model was used to generate synthetic‐CT images from MR images for each fraction. The synthetic‐CTs were independently aligned to the corresponding simulation‐CTs by seven physicists using rigid registration. The difference between CTgRT and MRgRT alignment was calculated to quantify the inter‐fractional image‐guidance uncertainty and estimate PTV margins. Dose from the clinical beam sets was recalculated on the synthetic‐CTs for comparative analysis.

**Results:**

The CTgRT alignment compared to MRgRT were −0.59 ± 3.49 mm, 2.04 ± 3.96 mm, and 1.23 ± 1.20 mm in left‐right (LR), superior‐inferior (SI), and anterior‐posterior (AP) directions, respectively. Compared to clinical plans, the dosimetric parameters of recalculated synthetic‐CT plans, V95 and D95, were significantly lower, while the homogeneity index was significantly higher (*p* < 0.001 for each metric). Compared to MRgRT, CTgRT required additional treatment margins of 10.0 mm in LR, 11.5 mm in SI, and 4.6 mm in AP directions.

**Conclusions:**

We quantified the dosimetric difference in treating intramuscular metastases using MRgRT versus CTgRT and showed that treatment margin can be reduced by at least 5 mm in each direction with MRgRT.

## 1 INTRODUCTION

Oligometastatic disease (OMD), which is widely considered to be an intermediate state between localized and systemic disease, is classified by the presence of 1–5 metastatic lesions that can each be safely treated with local therapy.^1^, ^2^ Stereotactic ablative radiotherapy (SABR, also known as stereotactic body radiation therapy or SBRT) is one potential treatment strategy for OMD and has been investigated in several multi‐institutional trials.^3^, ^4^, ^5^, ^6^ Common findings in these trials are overall high rates of local‐recurrence free survival for lesions treated with SABR, resulting in improved progression‐free survival and low rates of severe toxicity for patients with OMD, including for those patients with radioresistant histology such as renal cell carcinoma.^7^ Two recent prospective trials investigating the use of SABR in lieu of systemic therapy for such cases have demonstrated similar success.^8^, ^9^

**FIGURE 1:**
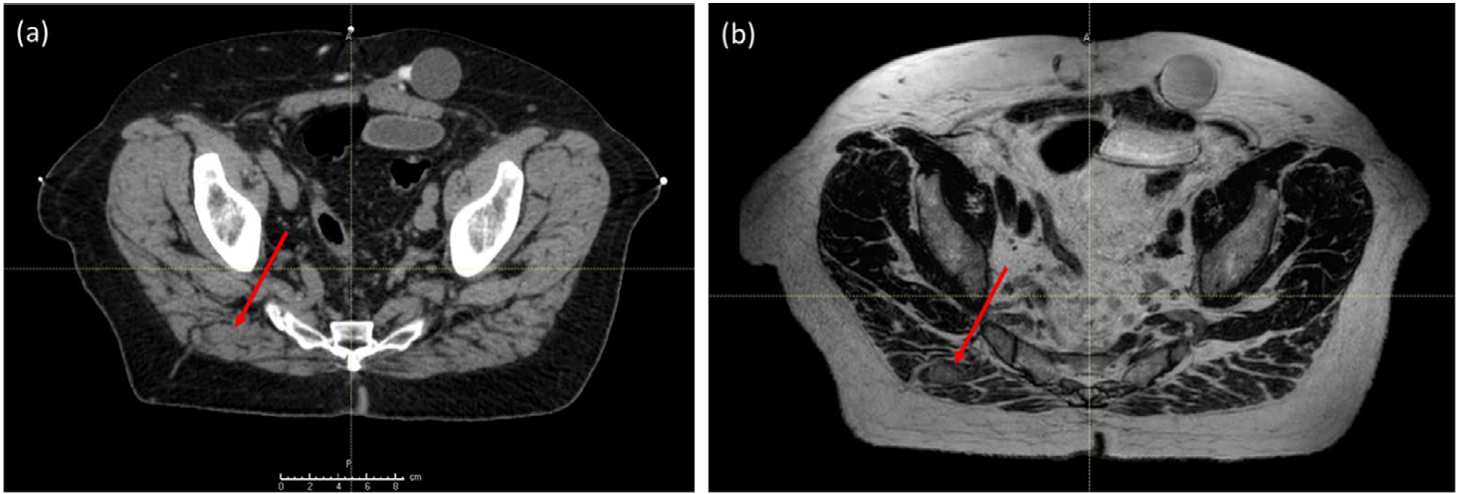
Transverse (a) CT and (b) MR images of a muscular metastasis. The tumor is visible on MRI but not on CT (red arrows).

Technologic advances in treatment setup imaging have greatly improved the accuracy of radiotherapy delivery, enabling image‐guided radiotherapy (IGRT), which is especially important for SABR because of its large dose gradients. On‐board x‐ray, cone‐beam computed tomography (CBCT), or CT‐on‐rails provide exquisite bony anatomy contrast, enabling CT‐guided radiotherapy (CTgRT) that can align lesions attached to bone, or with minimal motion relative to bone, with an accuracy of approximately 0.5–1.0 mm.^10^, ^11^ However, the alignment of lesions in soft tissue or thoracic or abdominal organs with low‐contrast on CT is much more difficult; it is particularly difficult for intramuscular lesions because the lesion displacement is not proportional to bone so that bony structures cannot be used as surrogate for alignment. Prior studies have shown that this can lead to setup errors of at least 1.5–5.0 mm and may require expansion of planning target volume (PTV) margins by 5–10 mm to account to recover target coverage.^11^, ^12^ The advent of MR‐linear accelerators (MRL), using “on‐board” magnetic resonance imaging (MRI) for MR‐guided radiotherapy (MRgRT), has successfully overcome this limitation. MRI generates sufficiently high image contrast in soft tissues^13^, ^14^ so that soft‐tissue lesions can be directly leveraged into improved setup accuracy and subsequent target coverage.^15^, ^16^ Figure 1 illustrates a metastatic lesion in muscle that is clearly visible on MRI but not on CT. Because OMD can appear in a wide range of anatomic sites, use of MRL may be well suited for SABR in a large proportion of cases where the metastatic sites lie in tissues of mesenchymal origin.

This study aimed to evaluate the potential setup errors of OMD patients that received clinical treatment on the MRL if they had instead been set up using CT/CBCT image guidance. Daily clinical MR images were used to create synthetic CT images to mimic what would have been used during the daily imaging in CTgRT. This facilitated a paired analysis between the two IGRT types, rather than analyzing separate patient populations that were treated either with CTgRT or MRgRT. To isolate CTgRT and MRgRT differences from the adaptive radiotherapy capabilities of the MRL, the plan parameters of CT‐guided “daily synthetic plans” were set identical to the MR‐guided “daily clinical plans” without any reoptimization.

The dosimetric impact of these setup errors was quantified by recalculating dose coverage metrics from the IGRT offset. Lastly, margin expansions were calculated that would facilitate maintained target coverage in the presence of these IGRT‐based setup errors. The results of this study demonstrate the dosimetric differences based solely on the type of IGRT used and should communicate the advantage of using MR‐guided treatments for these OMD cases.

## 2 METHODS

### 2.1 Patients

This study was approved by institution review board of MD Anderson Cancer Center (Protocol #: 2022‐0521). Data were collected from five patients with OMD who were treated with Elekta Unity MRL. Briefly, three patients had primary renal cell carcinoma with metastasis in the gluteal muscle (to be treated to 60 Gy in 15 fractions); one had primary melanoma with metastasis in the iliacus muscle (40 Gy in 5 fractions), and one primary had myxofibrosarcoma with metastasis in the paraspinal muscle (30 Gy in 3 fractions) (Table 1). The OMD sites in three of the patients abutted bone and lesions in the other two were at least 8 mm from the nearest bone. We defined distance to bone from target as “abutting bone” (0 mm) and “far from bone” (>0 mm). The rationale for considering the distance to bone was that bone is clearly visible on CT and is often used as a surrogate for image registration during patient setup. Clinical GTV‐to‐PTV margins ranged from 8 to 15 mm in each direction (average of 10 mm). In total, we extracted 53 daily plans (one per fraction) across all five patients. Two patients had multiple reference plans due to adapt‐to‐shape (ATS) workflows (described in detail in Section 2.4).

**TABLE 1:** Patient characteristics.

Patient	OMD site	Distance from bone	GTV volume	Prescription dose	Number of daily plans	Number of cumulative plans
1	Gluteal muscle	0 mm	265.0 cm^3^	60 Gy	15	5
2	Paraspinal muscle	0 mm	3.7 cm^3^	30 Gy	3	2
3	Iliacus muscle	0 mm	11.6 cm^3^	40 Gy	5	1
4	Gluteal muscle	20 mm	4.7 cm^3^	60 Gy	15	1
5	Gluteal muscle	8 mm	14.7 cm^3^	60 Gy	15	1

### 2.2 Simulation CT image acquisition

Each patient underwent CT simulation without contrast (Brilliance Big Bore, Philips Healthcare, Best, The Netherlands) to generate the clinical reference treatment plans. The simulation CT image properties were kVp = 120 kV, mAs = 400 mAs, FOV = 600 × 600 mm^2^, in‐plane resolution = 1.17 × 1.17 mm^2^, slice thickness = 2.5 mm, pitch = 0.813.

### 2.3 Simulation MR and daily MR image acquisition

Each patient also underwent MR simulation on a 1.5T MRL (Unity, Elekta AB, Stockholm, Sweden) on the same day following CT simulation, using the same setup positioning as the MR simulation. The planning image was a 3D T2‐weighted fast spin echo sequence with the following parameters: TR = 1300 ms, TE = 81.8 ms, NEX = 2, FOV = 400 × 448 mm^2^, in‐plane resolution = 0.52 × 0.52 mm^2^, slice thickness = 0.6 mm, bandwidth = 753 Hz/pixel. The same scan parameters were used to obtain daily images during each fraction for daily adaptation of the treatment plan.

### 2.4 MRL treatment workflow

**FIGURE 2:**
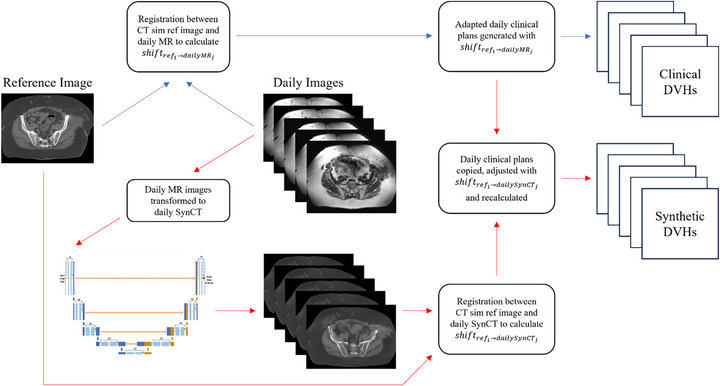
Magnetic resonance‐linear accelerator (MRL) treatment workflow. The clinical workflow is indicated by blue arrows; steps taken to generate daily synthetic plans are indicated by red arrows. CT, computed tomography; MR, magnetic resonance; DVH, dose‐volume histogram.

The workflow for daily clinical treatments on the Elekta Unity MR‐Linac are illustrated in Figure 2 (blue arrows). The daily MRI is registered to the reference CT image to determine the patient shift from planning to treatment. The reference plan is then adapted to account for this shift using either “Adapt‐to‐position” (ATP) or “Adapt‐to‐shape” (ATS) techniques. For this study, the choice of ATP versus ATS in the daily clinical plan did not matter‐ that is because the same plan parameters from the resultant “daily clinical plan” were also used for the daily synthetic plan (discussed below). Thus, the blue arrows in Figure 2 represent the general adaptive workflow, even though there are general differences between ATP and ATS plan generation. The only minor consequence of having the daily clinical plan result from an ATS workflow was that the daily MRI became the new reference image for subsequent daily clinical plans. This resulted in multiple cumulative plans for the same patient for dose‐volume histogram (DVH) analysis between the clinical and synthetic workflows.

### 2.5 Synthetic CT image generation

The generation of daily synthetic plans (Figure 2, red arrows) began with creating synthetic CT images from the daily MR images by using a previously published deep‐learning model.^17^, ^18^ The model utilizes a modified cycle‐consistent generative adversarial network (CycleGAN) architecture consisting of two generators and two discriminators. The generators employ a Residual‐UNet architecture, which integrates the feature preservation of a U‐Net with the gradient propagation benefits of residual blocks to perform image domain translation from MRI to CT. The discriminator employs a PatchGAN architecture to evaluate local image realism and is implemented as a stack of convolutional layers with progressively increasing feature channels with leaky ReLU activations, producing a sub‐regional realism score rather than a single global classification.

For this study, the model was trained using the T2‐weighted MR images and corresponding CT simulation images from 14 OMD patients. These MR and CT simulation images were pre‐processed and co‐registered to ensure anatomical correspondence. Images were resampled to a consistent spatial resolution and cropped to a uniform field‐of‐view for network input. MR intensities were normalized (z‐score normalization) to reduce inter‐scan intensity variability, and CT intensities were constrained to a clinically relevant Hounsfield Unit (HU) range before scaling for network training. Training optimized a weighted combination of adversarial loss, cycle‐consistency loss, and identity loss. The trained model was then used to generate synthetic CT images for the patients in the current study. As this model architecture has been extensively validated in prior works for both geometric fidelity and HU accuracy,^17^, ^18^ the generated synthetic CT images were visually checked to ensure anatomical consistency and image quality without further quantitative validation. Note that synthetic CT is a research tool for this type of analysis and is not currently used in routine clinical workflows for MRL treatments.

### 2.6 Synthetic CT image registration

Seven physicists (with experience ranging from 2 to 17 years and a median of 10 years) used RayStation treatment planning system (Raysearch Laboratories AB, Stockholm, Sweden) to rigidly register each of the daily synthetic CT images to the reference CT simulation image. Automated rigid registration, focusing on bone was initially performed, followed by manual adjustments. If the lesion was visible, the observers could register directly to it. Otherwise, they were asked to register the nearest bony structure. This rigid registration accounted for translational shifts only, without rotation, to simulate the setup and couch shift on a 3‐dimensional (IGRT) couch. Registration transformation values in the left/right (LR), superior/inferior (SI), and anterior/posterior (AP) directions were recorded, and the average transformation values were calculated across observers in each direction to determine the final CT‐guided registration transformation for each daily image. This registration transformation represents the alignment that would have been used for CTgRT.

### 2.7 Synthetic CT‐guided plan generation

Within the Monaco treatment planning system (TPS) environment (Elekta AB, Stockholm, Sweden), the daily clinical plans were perturbed to produce the daily synthetic plans according to the following workflow. The average CT‐guided registration transformation for the daily image, calculated in Section 2.6, was subtracted from the clinical MR‐guided registration transformation that was used to generate the daily clinical plan during treatment (Section 2.4). This is referred to as the registration offset, δ. The ATP workflow was initiated on the daily clinical plan (which was already adapted during the clinical treatment as described in Section 2.4). The clinical registration transformation was adjusted by δ, and the “Original Segments” setting was used to recalculate the dose distribution using the exact same beam parameters (including MLC control points and weighting) as the daily clinical plan. This resulted in the “daily synthetic plan”.

This workflow was carefully selected to ensure that the dosimetric differences between the daily clinical plan and daily synthetic plan were solely due to image registration differences. It can be conceptualized as an isocenter shift equivalent to δ and recalculation due to altered radiological path lengths, which is commonly utilized for robust planning evaluation. It should be emphasized that the beam sets and control points were identical between the daily clinical and synthetic plans. Both daily clinical and synthetic plans were calculated on the daily MR image in Monaco, just with differing registration transformations. The synthetic CT images were only used to calculate the registration transformation‐ the synthetic CT “HU values” were not used in any dose calculation in this study.

### 2.8 DVH curves and metrics calculations

Fractional dose‐volume histogram (DVH) curves for the gross tumor volume (GTV) were generated for each daily clinical and daily synthetic plan; these curves were calculated by rescaling the total adaptive plan dose to the dose delivered in 1 fraction. The following DVH metrics were analyzed: the volume (normalized to the GTV volume) covered by 95% of the prescription isodose (V95); the dose (normalized to the prescription dose) covering 95% of the target volume (D95); the maximum point dose (normalized to the prescription dose) in the target (Dmax); and the homogeneity index (HI), as defined by the International Commission on Radiation Units and Measurements in ICRU 83.^19^

$$\mathrm{HI}=\frac{D2-D98}{D_{Rx}}$$

where D2 and D98 are the doses (normalized to the prescription dose) covering 2% and 98% of the target volume, respectively. HI was included here to examine the effects of GTV dose homogeneity and uniformity when the registrations were shifted by using synthetic CT instead of MRI.

Daily plans associated with the same reference plan were summed together to produce cumulative DVH curves and associated DVH metrics. As mentioned in Section 2.4, daily clinical plans that were created using ATS resulted in separate cumulative DVH curves (one cumulative plan for each unique reference image). To avoid uncertainty, we did not perform deformable dose accumulation between multiple reference plans of the same patient.

### 2.9 Margin calculations

As mentioned in Section 2.7, the difference between the daily clinical and synthetic registration transformations is referred to as the registration offset, δ. These offset values were used to calculate recommended margins in each anatomic direction. The first step was to calculate Σ and σ, which are parameters in the classic van Herk formula that represent the standard deviations of systematic setup errors and random setup errors, respectively.^20^ These parameters can be calculated from a table of setup errors, $\delta_{i,j}$, using the following formulas, where *i* represents the number of fractions (1 to *P_j_*) and *j* represents the number of patients (1 to *Q*).

$$\;\mu_{j}=\frac{1}{P_{j}}\;\sum_{i=1}^{P_{j}}\delta_{i,j},\;S\;D_{j}^{2}=\frac{1}{P_{j}-1}\;\sum_{i=1}^{P_{j}}{\left(\delta_{i,j}-\mu_{j}\right)}^{2}$$

$$M=\frac{1}{Q}\;\sum_{j=1}^{Q}\mu_{j},\;\;\Sigma^{2}=\frac{1}{Q-1}\;\sum_{j=1}^{Q}{\left(\mu_{j}-M\right)}^{2},\;\;\sigma^{2}=\frac{1}{Q}\;\sum_{j=1}^{Q}SD_{j}^{2}$$

From this, $\Sigma_{eff}$ and $\sigma_{eff}$ were calculated to correct for the effects of hypofractionation according to the following formulas.^21^

$${\;\Sigma }_{eff}^{2}=\Sigma^{2}+\frac{\sigma^{2}}{N},\;\;\sigma_{eff}^{2}=\sigma^{2}\;\left(\frac{N-1}{N}\right)$$

where N was set to 15, which was equal to the most common fractionation scheme in our study. Then using the parameters α=2.5 and β=0.7, the Gordon and Siebers Hypofractionation formula was used to calculate the final margin values by using the following formula.^21^

$$\mathrm{Margin}=\alpha \Sigma_{eff}+\beta \sigma_{eff}$$

These α and β parameters estimate that 90% of patients receive complete GTV coverage of at least 95% of the prescription dose.

### 2.10 Statistical analysis

Analysis of the clinical and synthetic DVH metrics was split into subgroups: all fractional DVH metrics pooled together; fractional DVH metrics pooled by target location; all cumulative plan DVH metrics pooled together; and cumulative plan DVH metrics pooled by target location. For each analysis subgroup, individual two‐sided paired *t*‐tests were used to compare the clinical DVH metric with the corresponding synthetic DVH metric using GraphPad Prism version 9 (GraphPad Software, San Diego, California, USA). The resultant *p* values were adjusted for multiple comparisons by using Holm–Sidak formalism.

## 3 RESULTS

### 3.1 General DVH characteristics

**FIGURE 3:**
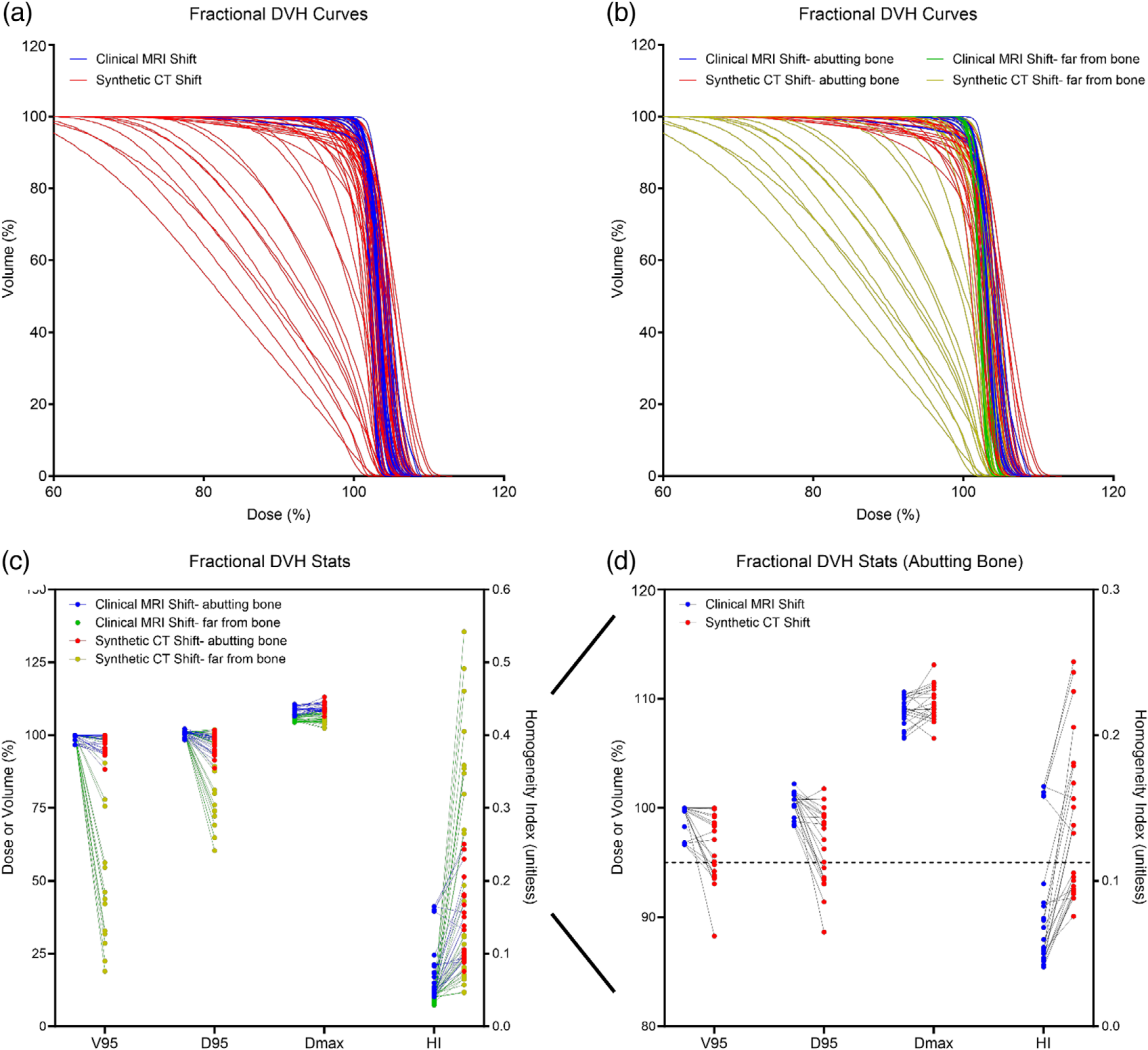
Fractional dose‐volume histogram (DVH) curves and statistics. DVH curves of the gross tumor volume (GTV) were created for the 53 daily plans generated with clinical MRI registration and with synthetic CT registration (a). The DVH curves were further grouped into “abutting bone” and “far‐from‐bone” groups based on the target location (b). DVH statistics were calculated from the DVH curves and compared pairwise between the clinical and synthetic plans (c). Each of these statistics still differed between clinical and synthetic plans in the “abutting bone” group (d).

Clinical and synthetic DVH curves of the GTV were created for the 53 daily clinical and daily synthetic plans (Figure 3a). Qualitatively, the shoulders of the daily synthetic DVH curves (shown in red) were broader and did not possess the sharp falloff that is typically found in clinically acceptable plans (shown in blue), which are indicative of high coverage of homogeneous dose. Compared to the daily clinical plans, the daily synthetic plans were characterized by significantly lower V95 (85.5 ± 24.4% vs. 99.6 ± 0.1%, *p* < 0.001) and D95 (92.4 ± 11.0% vs. 100.7 ± 0.8%, *p* < 0.001) values and significantly higher HI values (0.167 ± 0.121 vs. 0.057 ± 0.037, *p* < 0.001) (Table 2).

**TABLE 2:** DVH metric results.

	V95 (%)			D95 (% of Rx)			Dmax (% of Rx)			HI		
	Clin.	Syn.	*p*‐val	Clin.	Syn.	*p*‐val	Clin.	Syn.	*p*‐val	Clin.	Syn.	*p*‐val
Daily DVH: All plans	99.6 ± 1.0	85.5 ± 24.4	< 0.001	100.7 ± 0.8	92.4 ± 11.0	< 0.001	107.1 ± 1.9	107.7 + 2.4	0.004	0.057 ± 0.037	0.167 ± 0.121	< 0.001
Daily DVH: Plans abutting bone	99.2 ± 1.4	96.7 ± 3.2	0.002	100.5 ± 1.1	96.6 ± 3.4	< 0.001	108.9 ± 1.3	109.5 ± 1.5	0.052	0.081 ± 0.046	0.139 ± 0.054	< 0.001
Daily DVH: Plans far‐from‐bone	100.0 ± 0.0	77.0 ± 29.7	< 0.001	100.9 ± 0.3	89.2 ± 13.5	< 0.001	105.7 ± 0.9	106.2 ± 1.8	0.034	0.039 ± 0.005	0.188 ± 0.152	< 0.001
Cumulative DVH: All plans	99.5 ± 1.1	92.5 ± 16.9	0.406	100.9 ± 1.0	96.2 ± 6.3	0.120	107.5 ± 2.1	108.1 ± 2.6	0.406	0.066 ± 0.039	0.135 ± 0.065	0.011
Cumulative DVH: Plans abutting bone	99.3 ± 1.2	97.5 ± 2.2	0.051	100.8 ± 1.1	97.8 ± 2.3	0.017	108.4 ± 1.4	109.0 ± 1.9	0.335	0.076 ± 0.038	0.127 ± 0.040	< 0.001

### 3.2 Differences in clinical and synthetic DVHs based on target location

To further investigate the wide distribution of the daily synthetic plans, the plans were considered in two groups: according to whether the target abutted bone (23 of the 53 daily plans) or was far from bone (30 of the 53 daily plans) (Figure 3b). Qualitatively, the DVH curves of the “far from bone” daily synthetic plans (yellow curves) resulted in the most significant broadening of GTV dose falloff compared to the daily clinical plans (green curves). The differences in the DVH metrics between the synthetic and clinical daily plans were also exacerbated in the “far‐from‐bone” group (yellow points in Figure 3c). Compared to daily clinical plans (green points in Figure 3c), the “far from bone” daily synthetic plans had significantly lower V95 (77.0 ± 29.7% vs. 100.0 ± 0.0%, *p* < 0.001) and D95 (89.2 ± 13.5% vs. 100.9 ± 0.3%, *p* < 0.001) and significantly higher HI values (0.188 ± 0.152 vs. 0.039 ± 0.005, *p* < 0.001) (Table 2). The DVH curves of the “abutting‐bone” daily synthetic plans (red curves in Figure 3b) were more comparable qualitatively to the daily clinical DVH curves (blue curves), However, eight of these 23 “abutting bone” daily synthetic plans still would not have achieved the clinical goals of V95 > 95% and D95 > 95% (dashed line in Figure 3d) that are typically evaluated during planning. While to less extent as the “far‐from‐bone” plans, the “abutting bone” daily synthetic plans (red points) had significantly lower V95 (96.7 ± 3.2% vs. 99.2 ± 1.4%, *p* = 0.002) and D95 (96.6 ± 3.4% vs. 100.5 ± 1.1%, *p* < 0.001) and significantly higher HI values (0.139 ± 0.054 vs. 0.081 ± 0.046, *p* < 0.001) than the “abutting bone” daily clinical plans (blue points in Figure 3d, Table 2).

### 3.3 Differences in clinical and synthetic DVHs based on individual patient

**FIGURE 4:**
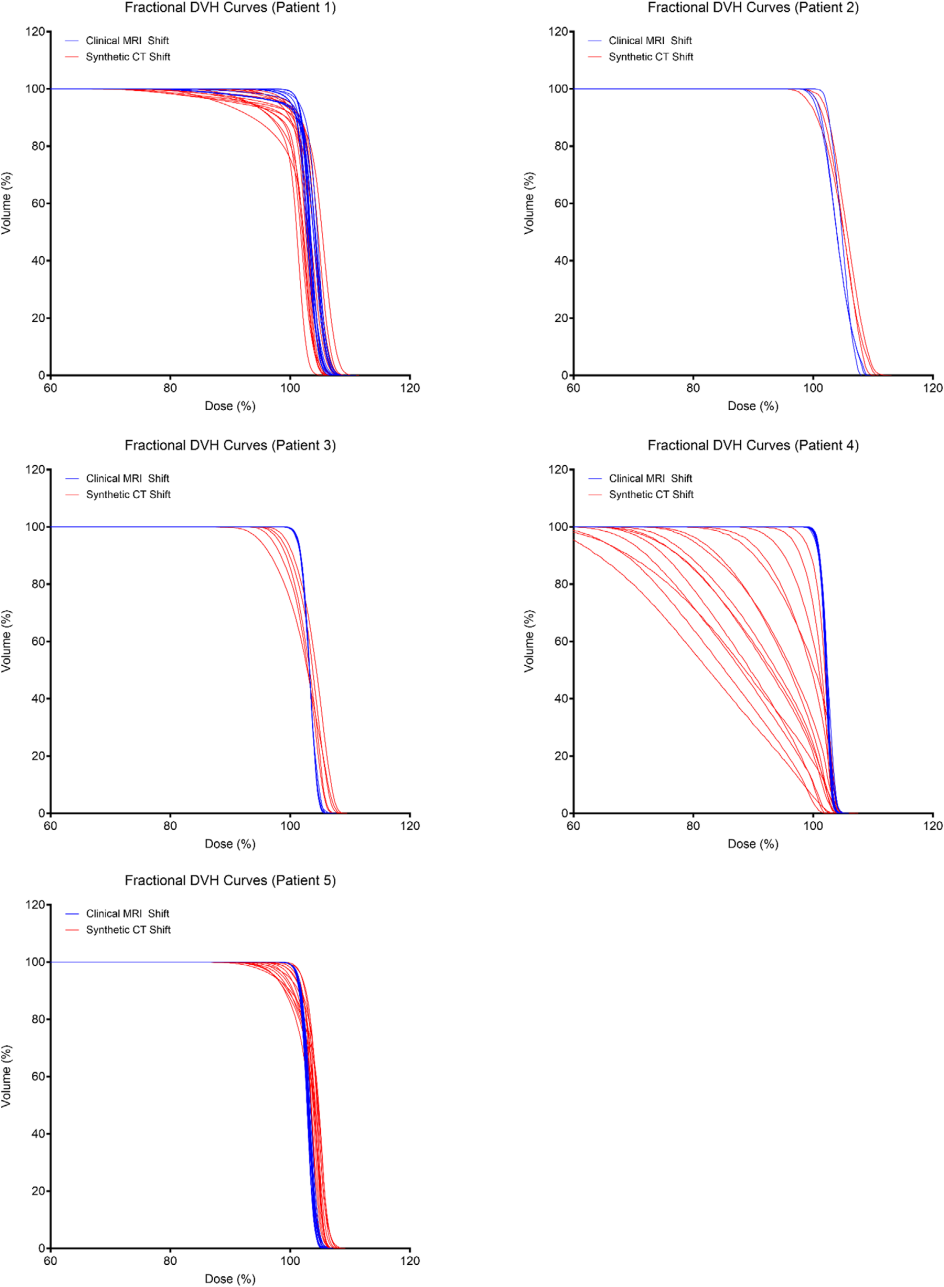
Fractional dose‐volume histogram (DVH) curves of the individual patients. The 53 fractional DVH curves were grouped according to the individual patient. The synthetic DVH curves were generally characterized as being more spread out with a less sharp falloff compared to the clinical DVH curves. These characteristics were exacerbated in Patient 4.

**FIGURE 5:**
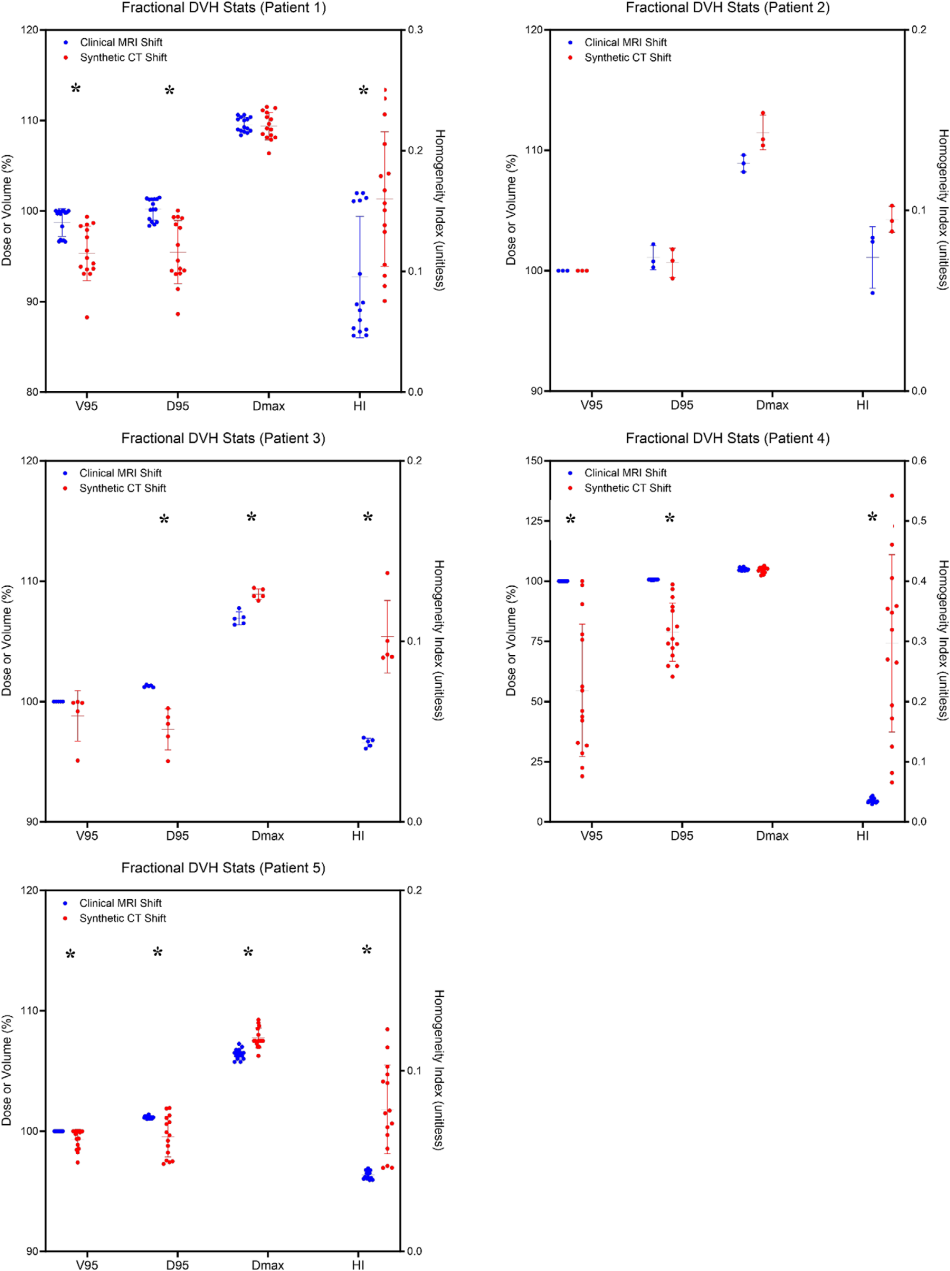
DVH metrics of the fractional dose‐volume histogram (DVH) curves of the individual patients. The V95, D95, Dmax, and homogeneity index (HI) values were compared between the clinical and synthetic plans for each patient. Only Patient 2 displayed no differences in any DVH metrics between the clinical and synthetic plans.

Graphs of the DVH curves were also plotted for each individual patient (Figure 4). Qualitatively the DVH curves of the daily synthetic plans (red curves) were characterized by a broader shoulder compared to the daily clinical plans (shown in blue) for each of the patients, but especially so in Patient 4 (which belonged to the “far from bone” group). DVH curves of Patient 3 (belonging to the “abutting bone” group) and Patient 5 (belonging to the “far from bone” group) also had noticeable shoulder broadening. The quantitative DVH metrics for each individual patient were also plotted (Figure 5). Compared to the daily clinical plans (blue points), daily synthetic plans (red points) possessed significantly worse D95 and HI in 4 of the patients and significantly worse V95 in 3 of the patients. In fact, only 1 of the 5 patients (Patient 2, who belonged to the “abutting bone” group) had no significant differences in DVH metrics between the daily clinical and daily synthetic plans.

### 3.4 Differences in cumulative clinical and synthetic plan DVHs

**FIGURE 6:**
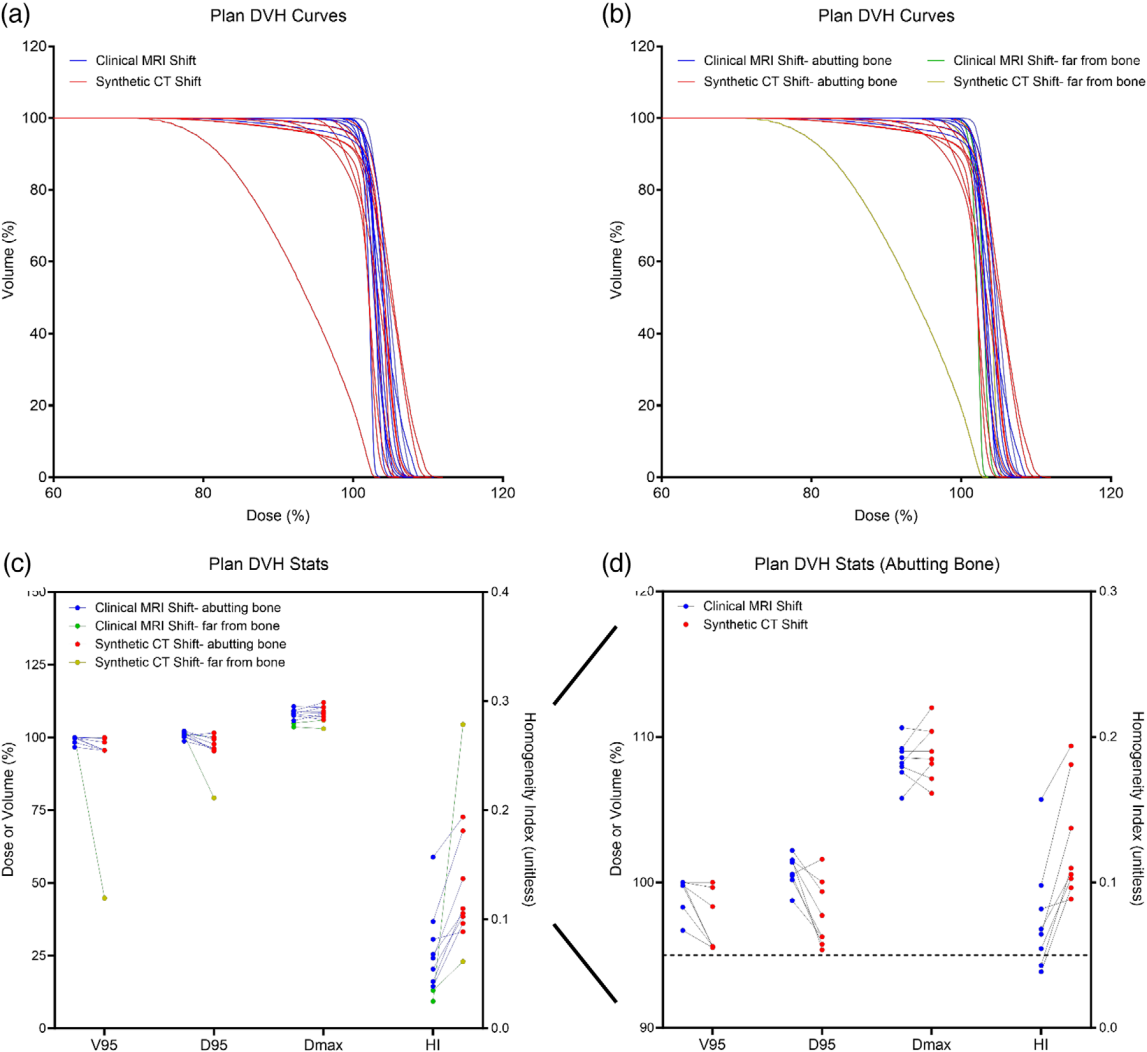
Cumulative plan dose‐volume histogram (DVH) curves and statistics. Daily plans that shared the same reference plan were summed together to generate 10 cumulative clinical and synthetic plan DVH curves of the gross tumor volume (GTV) (a). The DVH curves were further grouped into “abutting bone” and “far‐from‐bone” groups based on the target location (b). DVH statistics were calculated from the cumulative DVH curves and compared pairwise between clinical and synthetic plans (c). D95 and homogeneity index (HI) DVH statistics still differed between clinical and synthetic plans in the “abutting bone” group (d).

Daily plans that shared the same reference plan were summed together to generate cumulative clinical and synthetic plan DVH curves and associated metrics (Figure 6). Across the five patient cases, 10 cumulative plans were generated (the two patient cases with multiple ATS reference plans required multiple cumulative plans, as described in Methods). Data in Figure 6 is organized in the same manner as Figure 4. Qualitatively, cumulative synthetic plan DVH curves (red curves in Figure 6A) more closely represented cumulative clinical DVH curves (blue curves). Figure 6B demonstrates that the one exception to this belonged to a cumulative synthetic plan that was “far from bone” (yellow curve). This cumulative synthetic plan produced noticeable differences in the DVH metrics (yellow points in Figure 6C), though only average HI was significantly different (0.135 ± 0.065 vs. 0.066 ± 0.039, *p* = 0.011) across the cumulative synthetic plans compared to the cumulative clinical plans (Table 2). Of the 10 cumulative plans, 8 were “abutting bone”. When focusing on these cases, D95 (97.8 ± 2.3% vs. 100.8 ± 1.1%, *p* = 0.017) and HI (0.127 ± 0.040 vs. 0.076 ± 0.038, *p* < 0.001) were significantly different in the cumulative synthetic DVH plans (red points in Figure 6D) compared to the cumulative clinical DVH curves (blue points). Since there were only two “far from bone” cumulative plans, we could not run statistical analysis of the DVH metrics.

### 3.5 Margin calculations from CT registration offsets

The registration offsets resulting from the discrepancies between clinical MRI and synthetic CT registrations are tabulated in Table 3. The average registration offset values between the reference image and either the clinical MRI and synthetic CT were −0.59 ± 3.49 mm, 2.04 ± 3.96 mm and 1.23 ± 1.20 mm in the LR, SI, and AP directions, respectively. The resultant margins calculated from these offsets were 10.0 mm LR, 11.5 mm SI, and 4.6 mm AP. When considering just the three “abutting bone” cases, these values were 5.4 mm LR, 13.0 mm SI, and 4.3 mm AP. Reliable margin calculations could not be achieved for the two “far‐from‐bone” cases. As an assessment of interobserver variability with regards to synthetic CT image registration, the standard deviations between the observers across all registrations varied from 0.2 to 8.1 mm, and 96% of registrations had a standard deviation < 5.0 mm.

**TABLE 3:** Shift offsets between clinical MRI registration and synthetic CT registration and margin calculations.

	L/R Shift Offset (mm)					S/I Shift Offset (mm)					A/P Shift Offset (mm)				
Fx	Pat 1	Pat 2	Pat 3	Pat 4	Pat 5	Pat 1	Pat 2	Pat 3	Pat 4	Pat 5	Pat 1	Pat 2	Pat 3	Pat 4	Pat 5
1	7.40	1.91	−1.35	−7.24	−0.45	−0.89	−0.66	5.25	6.36	0.60	2.77	0.39	1.66	3.73	1.30
2	2.83	0.33	0.03	−2.95	0.97	−2.08	−1.91	7.97	0.00	0.63	3.63	0.21	2.97	1.66	0.83
3	2.15	−1.55	0.05	−9.13	2.35	−0.78	−5.75	5.63	10.3	0.25	2.89	−1.13	0.74	2.55	0.72
4	3.22		0.47	−3.23	0.86	6.29		6.60	3.05	0.08	7.73		0.56	−0.54	−0.18
5	4.28		−0.05	−3.88	0.66	2.48		5.03	5.84	−0.14	5.96		1.95	2.34	4.09
6	2.02			−3.81	−0.42	−1.60			5.28	4.44	−2.93			1.94	3.05
7	2.29			−3.45	0.37	−0.51			4.23	1.34	−0.17			0.95	3.19
8	2.34			−6.09	2.59	−1.99			8.47	−0.34	−1.50			2.37	3.32
9	0.68			−6.70	−0.33	0.72			4.99	−0.20	−2.60			3.37	2.62
10	−0.09			−9.66	−0.26	0.37			6.25	2.70	−5.20			4.52	3.87
11	2.40			−5.87	−2.44	−3.44			7.82	1.87	−2.81			−0.90	3.83
12	3.34			−8.90	0.49	1.58			4.72	1.01	−0.73			3.66	1.88
13	1.82			−10.6	0.62	−1.77			5.29	1.46	−2.12			3.10	2.24
14	5.18			−5.09	0.51	−1.54			11.2	3.72	−0.11			1.79	2.40
15	4.82			−9.76	0.78	−1.41			8.30	−1.42	−3.25			2.71	3.10
	**R/L**	**S/I**	**A/P**												
Offset Mean (M)	−0.59	2.04	1.23												
Offset SD (Σ)	3.49	3.96	1.20												
Offset RMS (σ)	1.76	2.22	1.96												
Effective Σ	3.51	4.00	1.30												
Effective σ	1.70	2.14	1.90												
Margin (mm)	9.98	11.51	4.58												

Abbreviations: A/P, anterior/posterior; L/R, left/right; RMS, root squares mean; S/I, superior/inferior; SD, standard deviation.

## 4 DISCUSSION

This study considered a group of 5 patients with OMD treated with an MRL and simulated the changes in image‐guidance and resultant dose distributions if CT‐guided setup were used instead of MRI. The resultant daily synthetic dose distributions indicated consistent reductions in V95 and D95 DVH parameters as well as increases in Dmax and HI compared with the clinical MRgRT plans. These results were more pronounced in OMD lesions that were not directly abutting bone. Cumulative plans, created by summing daily plans from the same reference image, did not produce as large a difference in dose statistics as the daily plans, with only the HI being significantly different between the synthetic and clinical plans. Finally, the offsets in image‐guidance registrations, used to calculate the clinical and synthetic dose distributions, were also input into the Gordon and Siebers hypofractionation margin formula,^21^ which suggested that additional margin values of 5–10 mm in each direction would have been needed to account for alignment errors purely due to lack of lesion conspicuity in the CT images.

With the daily clinical plan dose distribution positioned directly on the GTV during each fraction, registration offsets when utilizing the synthetic CT images caused corresponding shifts in the dose distribution around the GTV (as well as slight changes in dosimetry from differences in field depth). While this is typically accounted for with the planning tumor volume (PTV), large enough deviations cause parts of the GTV to fall outside the penumbra of the high dose region, which was observed in several of the daily synthetic plans as evidenced by the corresponding DVH curves. This causes a decrease in V95, which is the volume covered by 95% of the prescription dose. Correspondingly, D95, the minimum dose received by 95% of the volume also decreases due to the average of lower isodoses in part of the GTV. HI is a measure of how uniform the dose is within the GTV contour. A low HI indicates that a high proportion of the GTV is receiving the same dose (ideally the prescription dose). Shifting the GTV into lower isodose regions would result in a higher HI, as seen in many of the daily synthetic DVH curves. It's worth noting that higher HI values can sometimes be sought after in SABR plans, where there is a hot spot in the middle of the target. However, that is not the source of higher HI values in the synthetic plans in this study. Dmax was also measured in this analysis and included in the results, but as one might expect, there was not a substantial change due to CT registration shifts. Only Patient 2 had no significant differences in these DVH metrics, which is likely attributed to its small size (3.7 cm^3^) relative to the PTV margin (5 mm). Its motion relative to bone may also be more rigid due to it being in the paraspinal region rather than the pelvic region.

**FIGURE 7:**
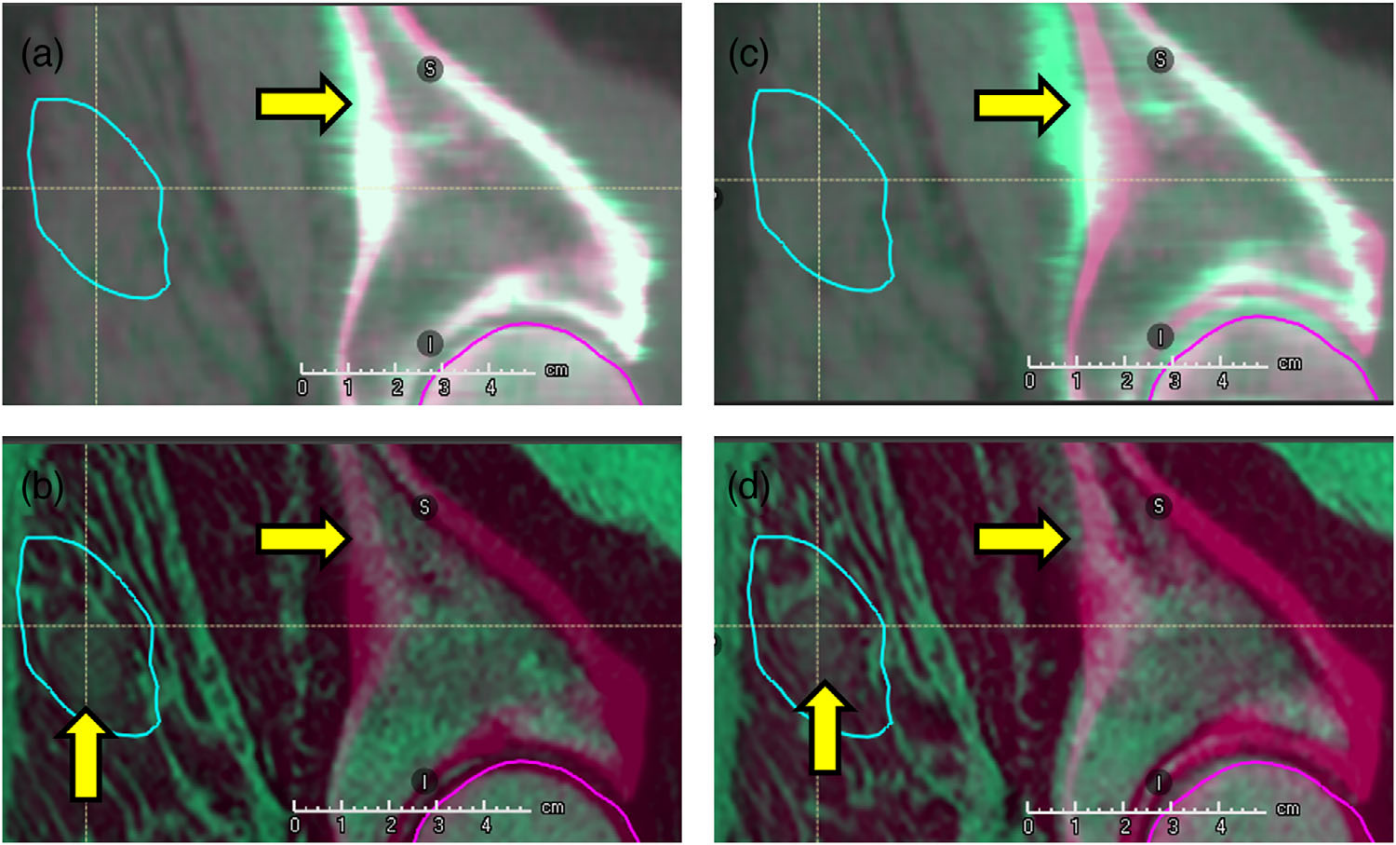
Example of CTgRT misalignment from Patient 4. When aligning the daily synthetic CT to the reference CT (a), the lesion is not visible, but the bony structures and external contour can be aligned. When applying this registration transformation to the daily clinical MRI (b), it is apparent that the lesion is not centered within the PTV and resides outside of the higher dose GTV (contour not shown for lesion visibility). When the daily clinical registration transformation is applied to the daily synthetic CT (c), the bony structures and external contour are misaligned, and the lesion is still not visible. When applying this registration transformation to the daily clinical MRI (d), the lesion is centered appropriately within the PTV (and GTV) contours.

Although individual fractions demonstrated significant deviations in DVH statistics when using the registrations from the synthetic CT images, cumulative plan quality was not significantly affected. This is consistent with the theory that random setup errors spread the dose out, rather than systematically shifting it. This works in the favor of plans with many fractions. However, in plans with few fractions, such as SABR plans, which are commonly prescribed for OMD, there is a higher risk of random errors not evenly spreading out, which would result in large deviations in plan quality between the anticipated and delivered plans. We also noticed that plans with targets far from bone were more susceptible to large deviations in image‐registration and resultant DVH statistics. Although our sample size (2 patients with 30 fractions) was not sufficient to compare “far‐from‐bone” cumulative plan DVH statistics between the synthetic and clinical plans, clearly at least one patient case (patient 4, with lesion 20 mm from bone) was noticeably affected (Figure 6A,B). To investigate whether this result could be attributed to erroneous registration by an observer, we calculated the root‐mean‐square error (RMSE) between each observer's synthetic shift and the average synthetic shift. However, omitting observers with large RMSE values did not result in improved DVH statistics. Thus, we believe that the source of lesion misalignment was due to significant non‐rigid motion between the lesion and bone between each fraction. This can occur due to soft tissue compression and deformation, which can be difficult to reproduce during patient setup each day. These misalignments are difficult to catch in CTgRT due to the lack of image contrast between the lesion and surrounding muscle. This can be visualized in Figure 7, where the CT‐guided and MR‐guided alignments are shown for one of the treatment fractions of this patient. The lesion was not visible in the synthetic CT, so CT‐guided registration was facilitated by bony alignment. From the clinical MR images, it is apparent that this registration would have led to nearly a 1 cm offset in lesion alignment. In fact, only Patient 1 had a lesion that was visible on CT, which was due to its large size that caused it to bulge out of the embedded muscle. Furthermore, respiratory motion can lead to large intrafraction motion of the lesion. While not investigated in this study, cine imaging on MRL devices can be used for beam gating, further ensuring accurate treatment delivery.^22^

To account for misalignments with CTgRT due to lack of lesion conspicuity, we calculated the anticipated margins that would be necessary to achieve the same coverage goals that were realized in the MRgRT treatments. Thus, the MRgRT clinical registration transformations were designated as the “ground truth,” and any differences in the transformation in the synthetic CT registrations were classified as setup errors (Table 3). The large variation of setup errors for a given patient across their treatment fractions indicates that accurate GTV alignment is not a rigid offset from the bone alignment. Thus, GTV delineation on a pretreatment MRI and registration with the simulation CT would not guarantee accurate alignment when using CTgRT. The classic van Herk formalism was applied to the tabulated setup errors, with a modification to account for the effects of hypofractionation.^20^, ^21^ The resultant margin values of 5–10 mm indicated the amount of additional irradiation volume that would have been required to reliably cover the target with prescription dose when treating these OMD with a CT‐guided setup. Equivalent setup errors were reported by Guckenberger et al. and Goff et al. when using CBCT to align soft tissue targets.^11^, ^12^ These margins could be added in quadrature to margins from other sources of error, such as motion, to assess differences in total treated volume. For larger lesions, such as those from Patient 1, these margins may be unnecessarily high since displacement of the surrounding tissue can aid with registration, even if the lesion‐muscle interface is unclear.

Besides the image contrast differences between CT and MRI, CTgRT, and MRgRT have different workflows for refining setup following daily image acquisition. For CTgRT, the couch is translated to best represent the geometry of the original treatment plan, and the same plan is delivered each fraction. For MRgRT, the treatment plan is adapted to best represent the dosimetry of the original treatment plan, and the couch is in the same position for each fraction. With this in mind, the methods in this study were carefully chosen to best isolate dosimetric changes arising from differing setup image registrations between CT and MRI and ignore any potential dosimetric changes arising from plan adaptation; thus, for the generation of the daily synthetic plans following the synthetic CT registration, it was necessary to not implement any further plan reoptimization that typically occurs following a registration shift in the clinical MRgRT workflow. The same multileaf collimator positions and weights were used in the clinical and synthetic versions of the daily plans; the dosimetric differences between the plans manifested from differences in plan isocenter locations from the associated registrations. Geometric distortion does exist in MR images, but annual measurements verify that this is <1 mm when measured 20 cm from isocenter. The TPS also accounts for any deviations between MR and Linac isocenter positions.

Another difference of the MRgRT workflow from the CTgRT workflow is that the reference image and plan will change if an adapt‐to‐shape (ATS) plan adaptation is implemented during one or more fractions throughout treatment. Dose to a target can be summed between fractions that have the same reference image, but advanced dose accumulation methods are required to sum dose between fractions of differing reference images and is beyond the scope of this study. Thus, for the two cases in this study that had at least one ATS, the cumulative treatment plan had to be separated into multiple sub‐plans, whose cumulative DVH curves were representative of the fractions belonging to a single reference image. This is why 10 cumulative clinical and synthetic DVH curves were analyzed in this study of five patients.

Beyond setup accuracy, the MRgRT workflow also has some practical advantages in clinics. First, clear visualization of soft tissue lesions eliminates the need for invasive fiducial markers often required in CTgRT and allows for potential treatment margin reduction. Second, daily plan re‐optimization accounts for anatomy shift to ensure target coverage and normal tissue sparing with treatment table in still position. This allows for easy patient setup and eliminates the risk of collision, which is often a concern in treatment with a C‐arm linear accelerator. Third, simultaneous MR imaging and beam delivery allows real‐time true tracking of tumor with automated gating, achieving high‐precision radiation treatment.

**FIGURE 8:**
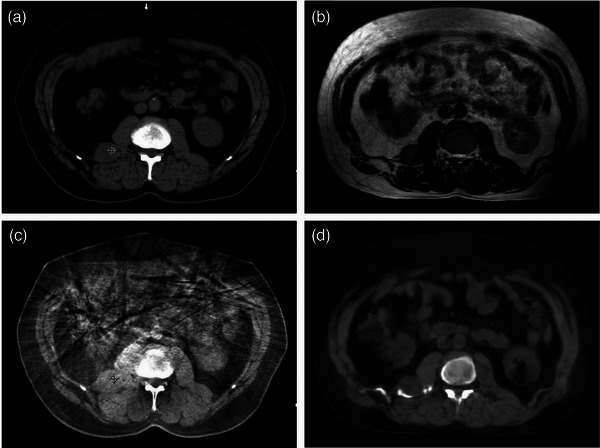
Image quality comparison between CT Simulation, MR Simulation, Daily CBCT, and Daily Synthetic CT. There is limited contrast between the lesion and adjacent muscle in the images from CT simulation (a) compared to the contrast in the images from MR Simulation (b). This contrast is worsened in daily CBCT images (c), which is further degraded by artifacts. Synthetic CT images (d), generated from MR images closely resemble those from CT simulation with regards to image quality and lesion contrast.

There are a few limitations of this study. One is the synthetic CT workflow not perfectly representative of a conventional CTgRT workflow, where the exact same reference plan is delivered in each fraction. However, we believe that this is as close of an approximation as achievable within the capabilities of the treatment planning system. This analysis likely even overestimates the accuracy of CT‐based registration since the image quality of the synthetic CT surpasses what is typically seen on CBCT (Figure 8). Rotations were also excluded as part of the multi‐observer synthetic CT registration, though 6 degree‐of‐freedom couch adjustments are now quite common in CTgRT and can help correct for patient rotation. Numerous reports have compared changes in dose distributions between plans treated on conventional CT‐guided Linacs and MRL, but those studies typically include the effects of adapting the treatment plan, resulting in nonequivalent comparison.^23^, ^24^, ^25^ While plan adaptation is a significant advantage of MRL‐based treatments, this study aimed to directly compare the margin variations resulting from tumor visibility on CT compared to MRI. Another limitation is the number of patients analyzed in this study. Average values for the dosimetry metrics from the synthetic plans may be biased by large discrepancies in a single patient. Furthermore, additional patients with lesions “far‐from‐bone” would enable cumulative plan comparisons in this subgroup, instead of being limited to the daily plan comparisons. While this may only provide an initial estimation of the dosimetric impacts of using CTgRT versus MRgRT, it should be noted that 53 individual treatment fractions across these patient treatments were analyzed. However, resultant margin calculations could be inflated due to large standard deviation values from a small sample size. We will increase the patient population with wider deviations in our future study.

## 5 CONCLUSIONS

This study demonstrated the practical advantage of MRgRT over CTgRT for the treatment of intramuscular OMD with SABR. These lesions are often small and embedded in soft tissue, which makes it difficult to align with CTgRT. Using a paired analysis between clinical MRL treatment plans and matched synthetic CTgRT treatment plans, these IGRT‐based alignments errors were shown to significantly reduce target coverage and homogeneity during each fraction, especially in lesions that were not abutting bone. This is impactful in hypofractionated regimens, where there are fewer opportunities to average out alignment errors. The calculated alignment errors between the clinical and synthetic daily plans were used to quantify margins of at least 5 mm that would have been necessary to maintain target coverage in CTgRT. As such, SABR treatment of OMD is substantially improved using MRgRT, even when only focusing on IGRT advantages. Future directions of this research will investigate further improvement of these treatments using the adaptive replanning and image‐guided gating capabilities of the MRL.

## AUTHOR CONTRIBUTIONS

Surendra Prajapati, Chad Tang, and Jinzhong Yang conceptualized the project. Travis Salzillo, Surendra Prajapati, and Jinzhong Yang contributed to data collection and analysis. Travis Salzillo drafted the manuscript with direct consultation with Surendra Prajapati and Jinzhong Yang. Shared first authorship is justified by the combined conceptual, analytic, and writing efforts of Travis Salzillo and Surendra Prajapati. Chad Tang contributed to clinical guidance and Jinzhong Yang contributed to technical guidance of project implementation. Their shared senior authorship is justified by the combined conceptual and project leadership efforts. Travis Salzillo, Surendra Prajapati, Z. Henry Yu, Tze Yee Lim, He Wanf, Xin A. Wang, and Rajat Kudchadker contributed to image registration and margin analysis. Yao Zhao and Xinru Chen contributed to synthetic Chad Tang model implementation and validation. Belinda Lee and Ahsan S. Farooqi contributed to treatment planning. Ahsan S. Farooqi, Devarati Mitra, Andrew Bishop, and Chad Tang contributed to clinical data collection and verification. All authors contributed to reviewing and revising the manuscript.

## CONFLICTS OF INTEREST STATEMENT

The authors declare no conflicts of interest

## ETHICS STATEMENT

This study was approved by institution review board of MD Anderson Cancer Center (Protocol #: 2022‐0521).

## Data Availability

Authors will share data upon request to the corresponding author.
